# Patient-derived xenografts as compatible models for precision oncology

**DOI:** 10.1186/s42826-020-00045-1

**Published:** 2020-05-20

**Authors:** Sung-Yup Cho

**Affiliations:** 1 Department of Biomedical Sciences, Seoul National University College of Medicine, 103 Daehak-ro, Jongno-gu, Seoul, 03080 South Korea; 2 Cancer Research Institute, Seoul National University College of Medicine, Seoul, South Korea; 3 Medical Research Center, Genomic Medicine Institute (GMI), Seoul National University, Seoul, South Korea

**Keywords:** Patient-derived xenograft, Precision oncology, Cancer preclinical model, Tumor heterogeneity, Targeted therapeutics, Drug efficacy test

## Abstract

Cancer is a very heterogeneous disease, displaying heterogeneity between patients (inter-tumoral heterogeneity) and heterogeneity within a patient (intra-tumoral heterogeneity). Precision oncology is a diagnostic and therapeutic approach for cancers based on the stratification of patients using genomic and molecular profiling of tumors. To develop diagnostic and therapeutic tools for the application of precision oncology, appropriate preclinical mouse models that reflect tumor heterogeneity are required. Patient-derived xenograft (PDX) models are generated by the engraftment of patient tumors into immunodeficient mice that retain several aspects of the patient’s tumor characteristics, including inter-tumoral heterogeneity and intra-tumoral heterogeneity. Therefore, PDX models can be applied in various developmental steps of cancer diagnostics and therapeutics, such as biomarker development, companion diagnostics, drug efficacy testing, overcoming drug resistance, and co-clinical trials. This review summarizes the diverse aspects of PDX models, addressing the factors considered for PDX generation, application of PDX models for cancer research, and future directions of PDX models.

## Introduction

Cancer is a heterogeneous disease caused by the combination of genomic, epigenomic, and transcriptomic alterations in cells. Recent studies using high-throughput parallel sequencing technology have demonstrated that even tumors from the same organ possess varying combinations of such alterations [1–3]. Therefore, to understand the natural cancer biology and provide appropriate interventions, precise stratification of cancers is required. Precision oncology is a strategic approach for cancer treatment, which uses molecular profiling of cancers for stratification of patients [4]. The strategy of precision oncology is closely associated with the development of biomarkers for cancer stratification and targeted therapeutics for the classified subgroups.

One of the hurdles for the development of biomarkers and targeted therapeutics is the availability of suitable preclinical animal models. Popularly used mouse models, including cell line-derived xenograft models and genetically engineered mouse (GEM) models, display only limited genetic diversity compared with the heterogeneous characteristics of human cancers [5]. Ideal animal models for cancer research should maintain the diversity of patient characteristics and allow for easy performance of model generation. Patient-derived xenograft (PDX) models are generated by transplanting the patient’s intact tumor tissue into immunodeficient mice, resulting in the growth of human cancers in the background of a mouse host [6]. As a result, a considerable degree of the patient’s tumor characteristics can be preserved, including histological and genomic features [7]. Therefore, of all the preclinical cancer models developed to date, PDX models most closely reflect patients’ tumor characteristics and, accordingly, patients’ responsiveness to treatment [7, 8]. Therefore, PDX models can apply to diverse fields of cancer research.

To apply PDXs models to several areas of cancer research, their characteristics and limitations need to be evaluated. In this review, we summarize several aspects of PDX models, including factors related to model generation, application to diverse cancer research fields, and approaches to overcome limitations.

### Factors related to the generation of PDX models

#### General procedure for PDX generation

**Fig. 1:**
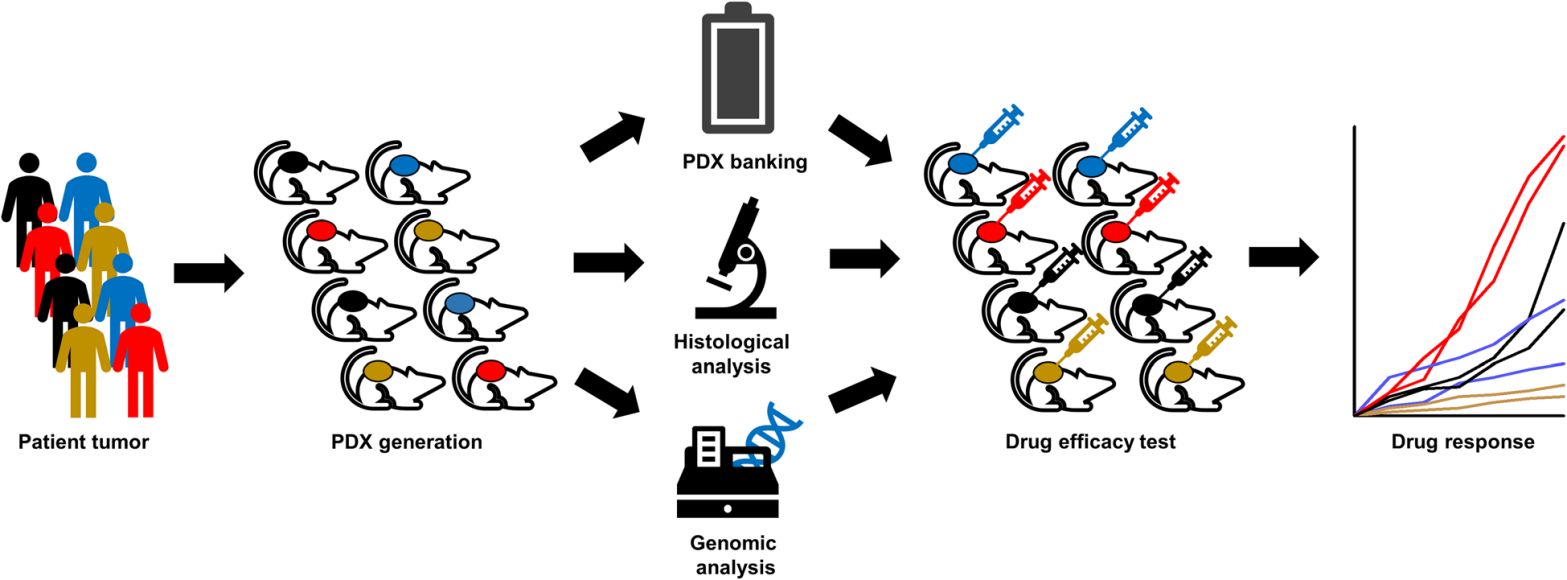
Scheme for generation of patient-derived xenograft (PDX) cohort

PDX models can be generated by engrafting patient tumor tissues into immunodeficient mice (Fig. 1). Tumor tissues are usually obtained from surgical specimens, but those obtained by biopsy can also be applied to generate models [9]. In the operation room, the obtained tumor tissues are washed and stored in cell culture media with antibiotics. To reduce tissue metabolism, they should be maintained at low temperature. Additionally, to reduce ischemia, the tumor tissues should be transferred to an animal facility as soon as possible. A prolonged ischemia time is known to be associated with a lower engraftment rate [10]. Tumor tissues are prepared by removing dead and necrotic areas and are then cut into sizes of 2–3 mm^3^. The mice are first prepared by anesthetization and sterilization of the implanting area. The tumor pieces are surgically implanted, usually in subcutaneous tissue. The compatible organs for the tumor origin can be considered for orthotopic implant models. After surgery, the mice are recovered from anesthesia by maintaining the body temperature using a warm pad or infrared lamp. Tumor growth is evaluated regularly, and when the tumor size reaches approximately 1000 mm^3^, the tumors are harvested and stored for the next passage. It usually takes about 3–6 months for tumor harvest, but it depends on the individual tumor characteristics [6]. The harvested tumors are prepared by removing necrotic areas and cutting into appropriate sizes for preservation. The PDX tumor tissues are stored in a liquid nitrogen tank by cryopreservation and can be re-implanted into new mice for the next passage. The generated PDX tumors are evaluated by histological and genomic analyses to ensure conservation of the patient’s tumor characteristics.

#### Tumor characteristics

The engraftment success rate of PDX models varies with the type of cohort reported and generally depends on the tissue types of tumor origin. Previous reports have demonstrated that colon cancers (76–89%) [11, 12], pancreatic cancers (45–62%) [13, 14], non-small cell lung cancers (41–90%) [15, 16], biliary tract cancers (54%) [17], head and neck cancers (85%) [18], and medulloblastoma (52%) [19] have high success rates of PDX generation. However, breast cancers (13–21%) [20, 21], gastric cancers (24%) [22], liver cancers (14%) [23], kidney cancers (37%) [24], bladder cancers (15%) [25], and melanoma (28%) [26] have low success rates (Table 1). The tumor histological types were reportedly associated with the success rate of PDX generation in some types of cancers, such as lung and gastric cancers (Table 1) [15, 22]. In the case of estrogen receptor-positive breast cancer with low malignancy, the success rate tends to be low; if necessary, the supplementation of human estrogen pellets would be required for model production [27]. Despite same tissue origin, tumors with clinically high malignancy or metastatic properties tend to exhibit a relatively high success rate [19, 28, 29]. In some reports, the success of PDX was associated with tumor aggressiveness, and patients whose tumors were successfully engrafted in the PDX models showed a poorer prognosis than those without success in PDX generation [30, 31]. Therefore, the success rate of the PDX models is largely affected by tumor origin and aggressiveness.

**Table 1:** Summary of engraftment rates of PDX tumors

Tumor type	Mice strain	Implantation site	Engraftment rate	Engraftment-related factors	References
Colorectal cancer	NSG	Subcutaneous	76%		Cho et al., 2019 [11]
	Nude	Orthotopic	89%		Aytes et al., 2012 [12]
Pancreatic cancer (ductal adenocarcinoma)	Nude	Subcutaneous	45%	Post-operation CA 19–9 level	Chen et al., 2020 [13]
	ICR SCID	Subcutaneous	67%		Mattie et al., 2013 [14]
Breast cancer	SCID/Beige, NSG	Mammary fat pad	19–21%		Zhang et al., 2013 [20]
	NOD/SCID	Humanized mammary fat pad	13%		Li et al., 2013 [21]
Non-small cell lung cancer	Nude, NOG	Subcutaneous	41%	Brain metastasis, SCC histology, tumor stage, wild-type EGFR	Lee et al., 2015 [15]
	NOD/SCID	Renal capsule	90%		Dong et al., 2010 [16]
Gastric cancer	NOG	Subcutaneous	24%	Intestinal type, high tumor cell percentage, short procedure time	Choi et al., 2016 [22]
Liver cancer (hepatocellular carcinoma)	NSG	Subcutaneous, orthotopic	14%		Zhu et al., 2020 [23]
Kidney cancer (renal cell carcinoma)	NOD/SCID	Orthotopic (Renal capsule)	37%	Tumor stage, Tumor implanted from metastatic site	Sivanand et al., 2012 [24]
Bladder cancer (urothelial carcinoma)	BALB/ c-nu	Subcutaneous	15%		Park et al., 2013 [25]
Biliary tract cancer (cholangiocarcinoma and gallbladder cancers)	NOD/SCID	Subcutaneous	54%	Surgical resection, median ischemic time	Leiting, 2020 [17]
Head and neck cancer (squamous cell carcinoma)	NSG	Subcutaneous	85%	Lymph node positive	Kimple et al., 2013 [18]
Medulloblastoma	Rag2 SCID	Orthotopic	52%		Zhao et al., 2012 [19]
Uveal melanoma	NOD/SCID	Subcutaneous	28%	Metastasis	Némati et al., 2010 [26]

#### Mouse strains

Several types of immunodeficient mice have been used to generate PDX models. Representative mouse types include nude mice, scid (severe combined immunodeficient) mice, non-obese diabetic (NOD)/scid mice, and NSG (NOD/scid/interleukin [IL] 2γ-receptor null) mice [6]. Because each mouse has different immunological characteristics and different degrees of immunosuppression in immune cell functions, it is important to understand the characteristics of each mouse strain and use compatible mice suited to the study purpose. Nude mice do not develop T cells because thymus development is inhibited by mutations in the *Foxn1* gene [32]. Scid mice lack T cells and B cells due to mutations in the *Prkdc* gene, which is involved in DNA double-strand break repair [33]. NOD/scid mice lack the functions of T cells, B cells, and natural killer (NK) cells [34]. NSG mice have an additional deletion of IL2γ receptors compared with NOD/scid mice. Therefore, these mice not only lack T cells, B cells, and NK cells just like NOD/scid mice, but also lack the function of immune cells related to innate immunity, such as macrophages and dendritic cells, resulting in the most severe immunosuppression among immunodeficient mice [35]. The success rate of PDX was reported to be lower in nude mice than in other types of mice because of the lower degree of immunosuppression, but no significant difference was reported in the success rates among the other types of mice [6]. The higher the degree of immunosuppression, the more likely the success rate of the PDX model; however, problems may arise due to the activation of human-derived viruses such as Epstein–Barr virus (EBV). Severe immunosuppressive mice such as NOD/scid and NSG mice have been reported to develop human cell-derived lymphoma caused by EBV activation of human cell origin [11, 36].

#### Transplantation sites

The most commonly used transplantation site for the generation of PDX models is the flank of the mouse (subcutaneous model; Table 2). The advantage of subcutaneous models is that the surgery required to generate the PDX model is very simple and tissue damage can be minimized. Thus, the mouse can easily recover after surgery. Additionally, because tumor growth can be directly evaluated through the skin, it is easy to confirm growth and measure the tumor volume change over time. However, the tumor characteristics become different from those of the primary tumor because the tumor grows in an environment different from that of the original organs [37, 38]. Additionally, subcutaneous models usually do not recapitulate the metastatic processes [37, 38]. Therefore, subcutaneous models can be considered first when constructing a large PDX cohort in a short time. The orthotopic model, in which tumors are transplanted according to the primary tumor site, attempted to overcome the limitations of the subcutaneous model (Table 2). Orthotopic models are produced by surgical transplantation of tumors in the same area as that of the primary tumor-derived organs. The most accessible orthotopic models are those for breast cancer because the mammary gland, the tissue from which breast cancer originates, is easily accessible from the outside and can be transplanted without major surgical procedures [39]. Orthotopic models can preserve the microenvironment characteristics of primary cancers because they are implanted in the organs of primary tumors and are more suitable for metastasis studies [37, 38]. However, skillful surgical techniques are required for successful implantation of tumor tissue. Additionally, because tumor growth is not usually detected from the outside, there is a limitation that monitoring tumor growth requires imaging such as ultrasound or computed tomography. The other option for tumor implantation is a subrenal capsule, which has the advantage of high blood vessel density, resulting in easy formation of blood vessels in tumor tissues (Table 2) [40]. This approach has been tried in several types of cancers, including prostate and ovarian cancers [41, 42]. Therefore, it is important to select a tumor transplantation site with the appropriate characteristics, according to the purpose of research.

**Table 2:** Comparison of several types of patient-derived xenograft models

PDX model	Advantage	Challenges
Subcutaneous model	• Easy procedure • Minimized tissue damage of mice • Easy evaluation of tumor growth • Maintaining tumor architecture and clonality	• Lack of proper tumor microenvironment • Lack of metastasis
Orthotopic model	• Preservation of microenvironment of primary tumor • Spontaneous metastasis	• Requirement of microsurgical skills • Imaging equipment required for longitudinal study
Subrenal model	• Increased blood supply for tumor growth	• Requirement of microsurgical skills • Imaging equipment required for longitudinal study
Humanized model	• Reconstitution of human immune cells • Evaluation of cancer immunotherapy	• Requirement of long time for humanization and PDX generation • Limited reconstitution of human immune system
Stromal cell co-implantation model	• Supply of human stromal cells in tumor microenvironment	• Change of tumor characteristics by stomal cells
Circulating tumor cell (CTC)-derived model	• Minimally invasive in patient • Easy to obtain samples • Applicable for otherwise unavailable tumor specimens • Preservation of intra-tumoral heterogeneity	• Requirement of technique for the enrichment of CTCs • Variable concentration of CTCs in blood

### Application of PDX models for cancer research

#### Cancer biology studies

A big advantage of the PDX models is that they retain much of the characteristics of the patients’ tumors [7, 8]. Therefore, various studies that are difficult to perform in patients can be achieved using PDX models. In cancer patients, invasive methods such as biopsies, which involve obtaining tumor tissues, are very limited in application. However, in PDX models, it is easy to acquire tumor tissue after various experimental treatments. Thus, PDX models can be applied to study diverse characteristics of tumor biology such as cancer growth, death, evolution, and metastasis.

PDX models retain the characteristics of the genomic mutations in patient tumors [11], so they can be applied to study the functions of cancer-related gene mutations. Comparing the characteristics of patient groups with or without a specific gene mutation can help assess the effect of that mutation on tumor biology. Analyses of tumor biological changes caused by gene mutations enable precision cancer medicine through the stratification of cancer patients according to their genome profile and the development of targeted treatment strategies [4].

Another area of ​​research where PDX models are applicable is ​​tumor heterogeneity. Tumors with the same pathology and originating from the same organs show inter-tumoral heterogeneity, manifesting as different histological, genetic, and malignant characteristics between patients, including morphology, gene expression, proliferation, invasion, and metastasis [43]. Additionally, tumors from the same patient demonstrate the characteristics of intra-tumoral heterogeneity, where histological, genetic, and malignant characteristics evolve according to chronological and regional changes in the tumor [44]. PDX cohorts generated from patient cohorts can be applied as a model to maintain inter-tumoral heterogeneity in patients [45]. Because the tissue from the patient is directly transplanted into the mouse in PDX models, various constituent cells existing in the tumor tissue can be transplanted together [11]. It is also possible to reflect changes in the genome due to cancer evolution, which can cause a considerable amount of heterogeneity in the tumor [11, 45]. Additionally, although a limitation of the mouse tumor microenvironment exists, PDX models recapitulate the pattern of in vivo cancer evolution; therefore, changes in the characteristics of cancer can be analyzed according to passage progression [45]. Thus, the PDX model can be used as an important tool to study cancer heterogeneity and evolution.

#### Biomarker development

To precisely diagnose and treat cancer patients, various biomarkers are required to evaluate the patient’s condition, and PDX models can be used as a tool to develop biomarkers for clinical application [39, 46]. PDX models make it easier to apply various experimental methods for tumor tissue analysis because patient tumor tissues can be additionally obtained through transplantation and expansion in mice. This is advantageous when only small amounts of biopsy samples are available for analyses. Using a large amount of tumor tissue, biomarker candidates for tumor diagnosis can be investigated using multi-omics analyses [47]. Additionally, biological samples other than tumor tissues, such as blood and urine, can be obtained from the PDX models, and biomarkers can be screened to diagnose tumors using proteomic and metabolomic analyses [48, 49].

The responsiveness to treatment in the PDX models is highly relevant for therapeutic responsiveness in patients [7, 8, 50]. Therefore, PDX models can be applied to develop biomarkers that predict therapeutic responsiveness. Rather than evaluating the therapeutic effect on the patient group directly, the PDX cohort can be treated and classified into responsive and non-responsive groups. The characteristics of two groups can be compared, which can provide biomarker candidates. In particular, PDX models offer the advantage of maintaining patient tumor characteristics and allow easy acquisition of biological samples, which can be applied to develop biomarkers [6, 46]. It is also possible to prospectively analyze PDX models to determine whether the biomarker is associated with the therapeutic effect; thus, PDX models can be used as a tool to validate biomarkers preclinically.

#### Evaluation of therapeutic efficacy

One of the largest obstacles for drug development in cancer chemotherapy is the limitation of models that precisely reflect the patient’s status. Although current drug development has been mainly carried out using cell line models, various studies have reported that the drug responsiveness in the cell line models does not sufficiently reflect in human patients. Cell line models cannot retain tumor heterogeneity and components of the tumor microenvironment present in the patients’ tumors [51], resulting in different treatment responsiveness. Previously used mouse models, including cell line xenograft models and GEM models of cancer-related genes, also have limitations in the validation of drug responsiveness because they do not exhibit the genetic diversity observed in cancer patients [5]. Thus, the effects seen in preclinical models may fail to be replicated in clinical trials, when these models are applied for the anti-cancer drug development process.

The PDX cohort has the advantage of being used as a model for genomic analyses while ensuring genetic diversity in cancer patients. Several previous reports have shown that drug responsiveness in PDX models is very similar to clinically applied drug responsiveness [6, 7, 50]. Therefore, evaluation of drug effectiveness in PDX cohorts can demonstrate the diversity of drug responsiveness in real patient groups. Additionally, it is possible to study the factors that determine drug responsiveness by identifying the responsive and non-responsive subgroups and analyzing their characteristics. Therefore, the PDX model is a valuable tool to validate anticancer drug effectiveness.

#### Drug resistance

Analyzing the responsiveness to specific drugs in the PDX cohort demonstrates the diversity of responsiveness observed in patients and allows the classification of responsive and non-responsive groups. The characteristics of the two subgroups can be compared using multi-omics analyses [11, 21, 52]. Specific characteristics of the non-responders identified using this approach can be used to develop biomarkers to predict drug responsiveness and can be applied to discover new targets applicable to combination therapy [52]. Prospective application of these results to PDX cohorts also allows validation of biomarkers or therapeutic targets [53].

#### Co-clinical trial

The final validation of the current drug development process occurs through clinical trials in patients. Clinical trials include phase 1 trials that prioritize toxicity assessments, phase 2 trials that evaluate the efficacy and titration of drugs in small-scale patients, and phase 3 clinical trials that evaluate the efficacy of drugs in large-scale patients [54]. However, because this process requires much time and money, efforts are being made to implement the clinical trial process more efficiently. One such approach is a co-clinical trial that involves both a clinical trial for patients and a preclinical trial using mouse models [55]. Concomitant with the clinical trials for patients, evaluation of drug responsiveness using PDX models can be used to obtain data on drug responsiveness, thus saving time and cost. Through this process, the protocol of clinical trials can be optimized. Additionally, analyses of the mouse models allow the simultaneous study of biomarkers related to drug responsiveness [56, 57], thus saving many resources required in the clinical trial process and increasing efficiency. Therefore, PDX models can be used at various stages of drug development for effective development of cancer therapeutics.

### Future directions

#### A genomically defined large PDX cohort

One of the suggested applications of PDX models is to adopt them as surrogates of matched cancer patients for drug responsiveness. By screening several chemotherapeutic drugs in PDX models, the most effective drug can be recommended prior to patient treatment. However, this approach is difficult to apply for conventional cancer treatment because the generation of PDX models is not successful in all patients and several months are required to obtain drug responsiveness data from PDX models [6]. Instead, the PDX cohorts with genomic data are a powerful tool for drug development. Because PDX models usually retain the genomic and transcriptomic characteristics of patient tumors, data from the screening of a specific drug can be further analyzed based on genomic profiles. A previous study demonstrated the feasibility of PDX clinical trials using approximately 1000 PDX models for 62 treatments and suggested several candidates for targeted therapy, biomarkers, and resistance mechanisms [52]. To utilize the PDX cohort for the drug development process, a large PDX cohort is required. Therefore, several consortium groups have tried to construct large PDX cohorts such as the EurOPDX consortium, the US National Cancer Institute repository of patient-derived models, and the Public Repository of Xenografts (Table 3) [38]. These genomically defined PDX cohorts can be applied for all aspects of drug development processes.

**Table 3:** Large cohorts of PDX consortiums

	Organization	Cancer types	No. of models	Genome data	Distribution	Web site
EurOPDX	EuroPDX consortium (16 academic institutions)	30	1924	Gene mutations	Available (Trans-national access or collaboration)	https://www.europdx.eu/
NCI Patient-Derived Models Repository (PDMR)	National Cancer Institute, USA	65	2925	OncoKB cancer panel, whole exome sequencing, RNA sequencing	Available	https://pdmr.cancer.gov/
Public Repository of Xenografts (PRoXe)	Weinstock Laboratory	Leukemia, lymphoma	157	Targeted exome sequencing (205 gene), RNA sequencing	Available	https://www.proxe.org/
The Jackson Laboratory PDX cohort	The Jackson Laboratory	35	400+	Targeted exome sequencing, whole exome sequencing, RNA sequencing	Available	https://www.jax.org/jax-mice-and-services/in-vivo-pharmacology/oncology-services/pdx-tumors
Novartis Institutes for Biomedical Research PDX Encyclopedia (NIBR PDXE)	Norvatis	16	1075	Whole exome sequencing, SNP array, RNA sequencing	Not available	https://www.nature.com/articles/nm.3954

#### Organoid-based PDXs

Patient-derived tumor organoids (PDTOs) are three-dimensional cell cultures derived from cancerous cells of patients’ tumor tissues [58]. PDTOs have been suggested as a preclinical model for precision oncology because they retain the genomic and histological characteristics of patients’ tumors [58, 59]. Compared with PDX models, PDTOs show a higher success rate and ease-of-use and can be applied for high-throughput screening [58, 59]. By contrast, PDX models preserve tumor heterogeneity and tumor-stromal interactions of patients’ tumors and are more relevant to study in vivo cancer biology and drug responsiveness [59]. Therefore, the integration of PDX and PDTO studies will bring more opportunities for precision oncology research. For example, PDX models can be established from PDTOs to increase the engraftment rate and PDTOs can be derived from PDX models for high-throughput drug screening. Additionally, combining drug responsiveness data from both models can increase the predictability of drug efficacy in clinical trials.

#### PDX models for the tumor microenvironment

Tumor tissue not only includes tumor cells but also non-malignant cells, including fibroblasts, immune cells, and endothelial cells. These surrounding cells interact with the tumor cells in various ways. The individual cells and their interactions inside the tumor tissue comprise the tumor microenvironment [60], which affects various phenotypes related to tumor biology. The tumor microenvironment has been reported to be associated with tumor aggressiveness, metastasis, and treatment responsiveness [61, 62]. Because PDX models generally use immunodeficient mice, limitations exist in studying the functions of immune cells in the tumor microenvironment. However, the tumor microenvironment can be studied in relation to the function of other cell types. When tumor tissues are transplanted into mice, non-malignant cells present in the tumor tissues are also transplanted together. Thus, in the case of an early-passage PDX model, there is a possibility to study the function of human-derived non-malignant cells. Analysis of the early-passage PDX model revealed that the extracellular matrix is maintained in about half of the PDX models, and in the remaining half, the extracellular matrix is replaced by mouse-derived proteins [11]. However, endothelial cells were mostly replaced by mouse-derived cells in all PDX models [11]; therefore, the use of PDX models may be limited in the case of antibodies targeting human-derived blood vessels. As the passage of the PDX model progresses, the proportion of human-derived cells decreases and the tumor microenvironment is gradually replaced by mouse-derived cells. Thus, it is necessary to considering these changes in the tumor microenvironment, while conducting experiments.

**Fig. 2:**
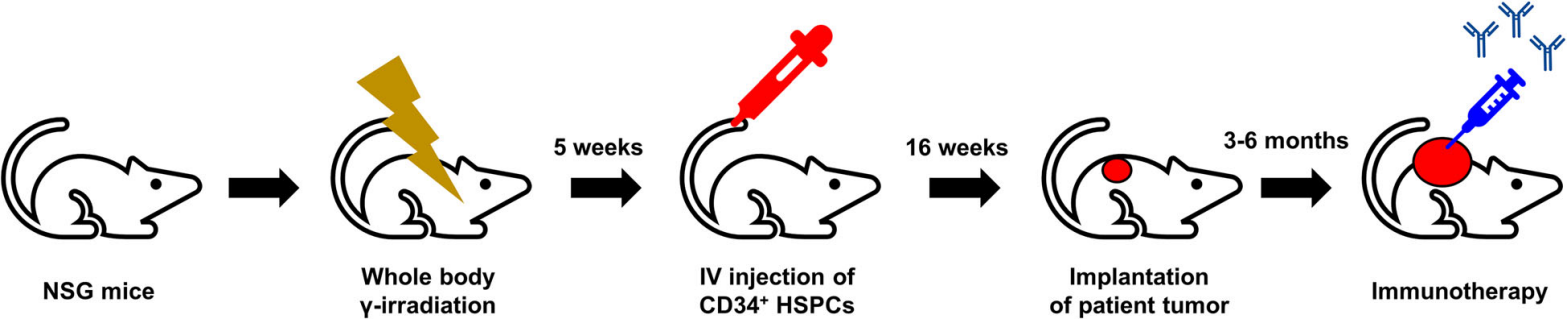
Scheme for generation of humanized patient-derived xenograft models. HSPC: hematopoietic stem and precursor cell

The development of several immune checkpoint inhibitors has opened new ways to cure cancers by harnessing the patient’s immunity [63]. In contrast to the success of cancer immunotherapy in clinics, the tools to investigate the preclinical effectiveness and underlying molecular mechanisms remain limited. Conventional PDX models are generated in immunodeficient mice; therefore, these models lack immune systems that resemble those of the host strains. To overcome these limitations, humanized mice, in which the immune cells are reconstituted by injecting CD34^+^ human hematopoietic stem and precursor cells [64], can be used to generate PDX models (Fig. 2; Table 2). The humanized PDX models can be established in partially human leukocyte antigen-matched allogenic immune systems. Infiltration of immune cells into tumors and tumor growth inhibition were observed with anti-PD1 therapy [65]. To improve the efficacy of the reconstitution of human immune cells, several transgenic or knock-in mouse models with human cytokines, such as macrophage colony-stimulating factor (CSF), IL3, granulocyte-macrophage-CSF, and stem cell factor, have been tried, especially to develop innate immune cells [66, 67]. The humanized PDX models are more relevant to study cancer biology, such as tumor structure, metastasis, and signaling, and can be a powerful tool to investigate cancer immunotherapy.

In the tumor microenvironment, stromal cells are highly associated with tumor aggressiveness, metastasis, and treatment responsiveness [61, 62]. Additionally, in PDX models, the components of the tumor microenvironment are steadily substituted by murine stroma as the xenografts are serially passaged [38]. To compensate for the human stromal component in PDX tumors, the strategy of co-engrafting human mesenchymal stem cells (MSCs) or cancer-associated fibroblasts with tumor tissue was investigated (Table 2) [45]. In breast cancer PDX models, implantation of human MSCs with tumors enhanced tumor growth and vascularization, preserving estrogen receptor expression [68]. However, the application of MSCs for PDX model generation needs to be carefully examined because the effect of MSCs in tumor development remains controversial [45].

#### Circulating tumor cell (CTC)-derived xenograft models

**Fig. 3:**
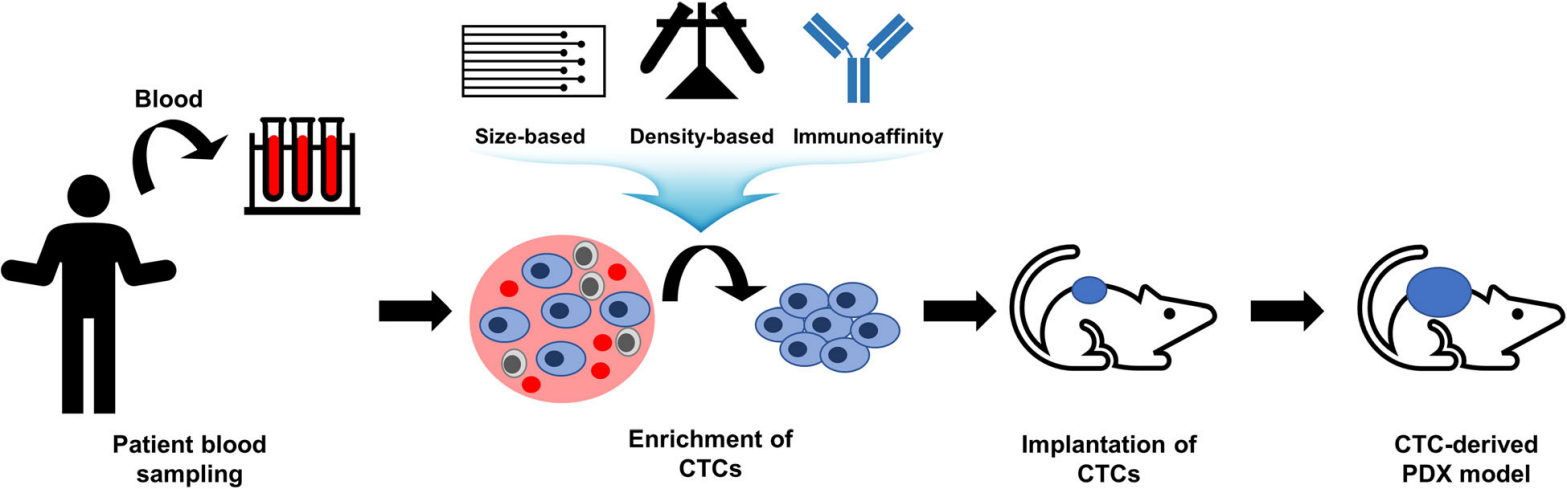
Scheme for generation of circulating tumor cell (CTC)-derived patient-derived xenograft (PDX) models

To establish PDX models, human tumor tissues need to be obtained by a surgical procedure or biopsy, which are very invasive methods for patients. For some types of cancers, it is almost impossible to obtain human tumor tissues. Additionally, a small fragment of tumor tissue is usually implanted for PDX generation, resulting in the loss of the intra-tumoral heterogeneity of whole human tumors. To overcome these limitations, CTC-derived xenograft models were attempted, in which the captured CTCs from patient blood samples were implanted into immunodeficient mice (Fig. 3; Table 2) [69, 70]. CTCs can be obtained from patient blood samples, which avoids the invasive procedure for patients [69, 70]. Additionally, all parts of the tumor comprise CTCs; therefore, CTCs are a mixed population of cancer cells that retain the intra-tumoral heterogeneity of patients [69, 70]. CTCs can be harvested using several capturing methods, including physical property-dependent enrichment using size and density differences and biological property-dependent enrichment using immunoaffinity capture [69]. In size-based separation, CTCs show a larger size and stiffness than blood cells; therefore, they can be enriched using membrane filters or microfluidic devices [71]. In density-based separation, CTCs are enriched based on the difference in the relative densities between CTCs and blood cells using density-based gradient centrifugation or isopycnic density gradient centrifugation [72]. Immunoaffinity-based CTC enrichment uses antibodies against CTC-specific surface markers such as EpCAM and other stem cell or mesenchymal markers [71]. In the small cell lung cancer cohort, from which tumor tissues are rarely obtainable, CTC-derived xenograft models showed considerable similarity of genomic characteristics to patients and mirrored the patients’ responsiveness to chemotherapy [73]. Additionally, CTC-derived xenografts were successfully established in several tumor types, including breast cancer, prostate cancer, gastric cancer, and melanoma [74–77].

#### Modified response evaluation criteria in solid tumors (RECIST)

In experiments using PDX models, tumor growth retardation compared with the control treatment group is usually regarded as effective. However, this evaluation does not always guarantee clinical effectiveness because, in this situation, the final tumor volume can be larger than the initial tumor volume. Therefore, quantitative metrics need to be adopted to evaluate the therapeutic effect more precisely [38]. In clinical situations, RECIST guidelines are prevalently used to assess treatment responsiveness [78]. Correspondingly, modified RECIST (mRECIST) was suggested to evaluate treatment responsiveness in PDX models [52]. mRECIST is estimated by the ‘Best Response’, which is defined as the minimum percent volume change for 10 days, and ‘Best Average Response’, which is defined as the minimum value of the average percent volume change for 10 days compared with the initial volume. Additionally, each criterion is described as follows: modified complete response (mCR), Best Response < − 95% and Best Average Response < − 40%; modified partial response (mPR), Best Response < − 50% and Best Average Response < − 20%; modified stable disease (mSD), Best Response < 35% and Best Average Response < 30%; modified progressive disease (mPD), not otherwise categorized [52]. For precise evaluation of treatment responsiveness, standardized and quantitative criteria need to be applied.

## Conclusions

Precision oncology requires detailed diagnostic stratification of patients based on genomic profiling and tailored therapeutics compatible with patients’ characteristics. Because PDX models are generated from patient tumor tissues, several aspects of human patient tumors including genomic and histological characteristics are readily preserved. Because of this advantage, PDX models provide a powerful route for several steps of the drug development process in precision oncology, such as drug efficacy testing, biomarker development, drug resistance studies, and co-clinical trials. When appropriately applied by weighing the pros and cons, PDX models are very useful to advance precision oncology.

## Data Availability

The datasets generated during the current study are available.

## Abbreviations

CAFs: Cancer-associated fibroblasts
CTC: Circulating tumor cell
EBV: Epstein–Barr virus
GEM: Genetically engineered mouse
HSPCs: Human hematopoietic stem and precursor cells
MSC: Mesenchymal stem cells
mCR: Modified complete response
mPD: Modified progressive disease
mPR: Modified partial response
mRECIST: Modified response evaluation criteria in solid tumors
mSD: Modified stable disease
NOD: Non-obese diabetic
NSG: NOD / scid / IL2γ-receptor null
PDX: Patient-derived xenograft
PRoXe: Public Repository of Xenografts
RECIST: Response evaluation criteria in solid tumors; scid: severe combined immuno-deficient
